# Importance of pesticides for lethal poisoning in India during 1999 to 2018: a systematic review

**DOI:** 10.1186/s12889-021-11156-2

**Published:** 2021-07-22

**Authors:** Ayanthi Karunarathne, Ashish Bhalla, Aastha Sethi, Uditha Perera, Michael Eddleston

**Affiliations:** 1 Centre for Pesticide Suicide Prevention, and Pharmacology, Toxicology & Therapeutics, University/BHF Centre for Cardiovascular Science, University of Edinburgh, Edinburgh, UK; 2 Department of Internal Medicine, Nehru Hospital, Institute of Medical Education and Research, Chandigarh, India; 3 Government of Maharashtra, Mumbai, Maharashtra India; 4 Usher Institute for Population Health Sciences & Informatics, University of Edinburgh, Edinburgh, UK

**Keywords:** Poisoning, Deaths, India, Aluminium phosphide, Pesticide

## Abstract

**Background:**

Poisoning is a major problem in India. However, there is little systematic information on the key poisons responsible for most deaths by geographical area and over time. We aimed to review the literature to identify the poison classes causing the greatest number of deaths in India over the last 20 years.

**Methods:**

We performed a systematic literature review in Medline, Embase and Google Scholar (1999–2018), and Indian online medical journals, to find papers that reported deaths from all forms of poisoning in India, with last search 20 April 2020. We included epidemiological studies, observational studies, randomised trials, interventional studies, and case series published from 1999 to 2018 that showed the number of deaths and autopsy studies indicating the specific poisons or poison classes. Studies providing the case fatality for specific poisons or classes, which enabled calculation of the number of deaths, were also included. We excluded deaths due to animal bites and stings, ethanol or methanol poisoning, and gas inhalation as well as papers reporting a single death (case study of single patient). We grouped the papers into 5-year intervals and identified the two most common poison classes in each paper. We used descriptive statistics to summarise the findings over time based on the causative poison and the location of the study.

**Results:**

We identified 186 papers reporting 16,659 poisoning deaths between 1999 and 2018. The number of publications per 5-year interval showed no clear trend over the period (48, 38, 67, and 36 for consecutive periods). Half of the deaths (*n* = 8338, 50.0%) were reported during the first 5 years of the study (1999–2003), the number of deaths declining thereafter (to *n* = 1714 in 2014–2018). Deaths due to pesticide poisoning (94.5%) were dominant across the study period compared to other classes of poison [hair dye paraphenylenediamine poisoning (2.6%), medicine overdose (1.4%) or plant poisoning (1.0%)]. Among the pesticides, aluminium phosphide was the most important lethal poison during the first 10 years before declining markedly; organophosphorus insecticides were important throughout the period, becoming dominant in the last decade as aluminium phosphide cases declined. Unfortunately, few papers identified the specific organophosphorus insecticide responsible for deaths.

**Conclusion:**

Use of the published literature to better understand the epidemiology of lethal poisoning in India has clear limitations, including secular variation in publishing practices and interest in poisoning. Unfortunately, there are no long-term detailed, combination hospital and community studies from India to provide this information. In their absence, our review indicates that pesticides are the most important poison in India, with organophosphorus insecticides replacing aluminium phosphide as the key lethal poison after government regulatory changes in 2001 reduced the latter’s lethality. Plant and hair dye poisoning and medicines overdose caused few deaths. Aluminium phosphide deaths mostly occurred in northern Indian states, whereas deaths from organophosphorus insecticide poisoning occurred throughout India. Paraquat poisoning has become a clinical problem in the last 10 years. Lethal pesticide poisoning remains alarmingly common, emphasising the need for additional regulatory interventions to curtail the burden of pesticide poisoning deaths in India. More detailed reporting about the specific pesticide involved in lethal poisoning will be helpful to guide regulatory decisions.

## Introduction

Poisoning is a global public health problem. The World Health Organisation (WHO) estimates that nearly 250,000 deaths occur globally due to poisoning each year, with pesticides alone causing 150,000 deaths [1]. Most poisoning deaths occur in lower- and middle-income countries (LMIC) [2]. The number of deaths occurring from particular poisons or poison classes varies from place to place, and over time, as a result of changes in access, effective and timely medical management, preventative measures, and regulatory policies.

Pesticide poisoning has been a major problem in India since at least 1958, when 100 people died after consuming flour contaminated with the organophosphorus (OP) insecticide parathion [3]. An estimated 230,000 suicides occur each year in India [4], of whom at least 70,000 (30%) die from pesticide suicide [5]. Plant poisoning is well recognised with deaths occurring from suicide, homicide, and unintentional poisoning [6]. Datura (*Datura metel*) was the poison of choice in homicide in nineteenth century [6] while other plants currently implicated in self-poisoning include *Cascabela thevetia*, *Cerbera manghas* or *odollam*, and *Cleistanthus collinis* [7] (likely due to easy availability [6]). Medicine overdose is a major problem in the United States of America and many European counties [8, 9] but its role in India is considered minor [7, 10].

The National Poisons Information Centre (PIC), one of several poison centres in India, provides information over the phone concerning the diagnosis, and management of poisoning [11–13]. These calls indicate that pesticides, medicines, and plants are important poisons used across the country. However, the number of PIC calls per year is relatively small compared to the population of India and does not give a representative picture of the situation across the country or identify the poisons killing most people.

Comprehensive longitudinal prospective case series from several hospitals across the country, such as has been done for Sri Lanka [14–16], would be the ideal sources for information on causes of poisoning death over time, but such studies are only available from Chandigarh (for example ref. [17, 18]). We therefore took a literature review approach, aiming to identify the most important poisons and poison classes for death in India over the last 20 years from the published literature.

## Methods

We performed a systematic review to identify papers reporting poisons or poison classes responsible for poisoning deaths in India over the last two decades (1999–2018).

Two authors (AK, AS) independently searched Medline, Embase and Google Scholar, using the terms in all fields ((((poison OR poisoning) NOT (bite OR alcohol OR methanol)) AND India) AND (death OR mortality)). We also searched online Indian journal websites for relevant papers, including the *Journal of Association of Physicians of India*, *Journal of Indian Academy of Forensic Medicine*, *Indian Journal of Paediatrics*, *Indian Journal of Public Health*, and *Journal of the Indian Medical Association*. We limited the search to English language publications or those with an English language summary. The databases were last searched on 20 April 2020.

We looked at all modes of poisoning, including unintentional, occupational and intentional, involving agrochemical and industrial chemicals, toxic plants, and medicine poison classes.

### Inclusion criteria

We included epidemiological studies, observational studies, randomised trials, interventional studies, and case series published from 1999 to 2018 that showed the number of deaths and autopsy studies indicating the specific poisons or poison classes. Studies providing the case fatality for specific poisons or classes, which enabled calculation of the number of deaths, were also included.

### Exclusion criteria

Deaths due to animal bites and stings, ethanol or methanol poisoning, and gas inhalation were excluded from this study as well as papers reporting a single death (case study of single patient). Animal bites were not included in the study because the epidemiology and intent of envenoming is quite different to poisoning, the subject of this paper. The precise cause of acute death with alcohol or methanol toxicity is often unclear. Death may occur from chronic effects of alcoholism, such as variceal bleeding and cirrhosis. We therefore excluded these causes of death.

We screened the papers (UP & AK), removed duplicates, grouped the papers into five-year periods (1999–2003, 2004–2008, 2009–2013, 2014–2018), and identified the two most common poisons or poison classes in each paper (typically responsible for > 90% of deaths). Papers reporting deaths for more than one study period were considered as separate publications for each of the study periods and the death numbers extracted for each study period. If the number of deaths for each year was not reported in the paper but for the entire duration of the study, we calculated the number of deaths for each of the relevant study periods. Papers that did not provide this information were excluded.

We looked at the number of papers reporting deaths from poisoning during each study period, extracted the two most common groups of poisons. We summarised the data using simple descriptive statistics according to the poisons causing the most deaths and grouped them as pesticide, medicine, plant or other poisoning. We used SPSS (version 24) for the analysis. We did not register the review or publish a protocol for this descriptive review of poisoning deaths in India.

## Results

**Fig. 1:**
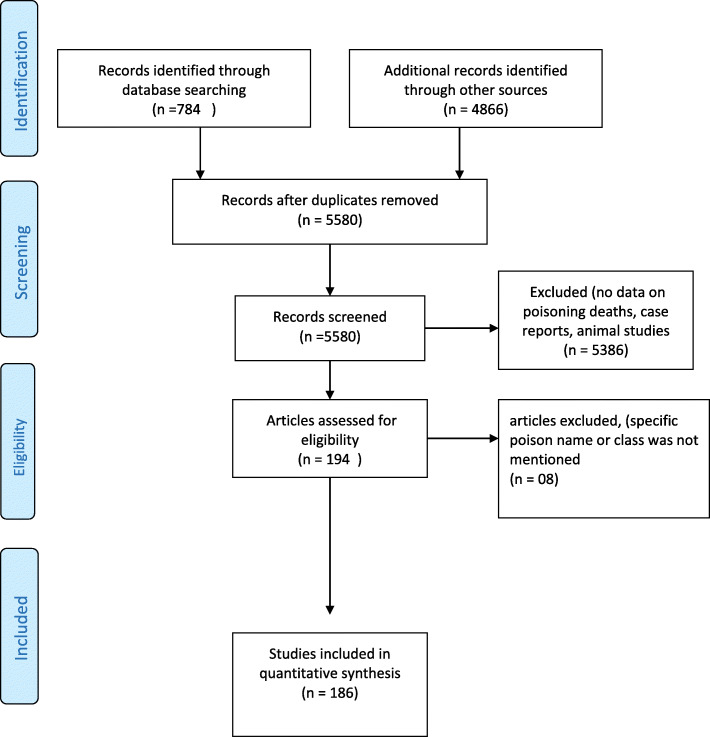
Flow diagram for data extraction

We identified 186 relevant publications reporting deaths from a specific poison or poison class in India during 1999 to 2018 (Fig. 1). The number of publications providing data for each 5-year interval showed no trend over the years: 1999–2003, *n* = 49 [17, 19–67]; 2004–2008, *n* = 38 [26, 65, 67–102]; 2009–2013, *n* = 67 [10, 65, 67, 100, 103–166]; and 2014–2018, *n* = 36 [167–202] (mean 48.2, SD 13.45).

There were 16,659 deaths from the two most important poison classes in each paper (Table 1). Half of the deaths reported (*n* = 8338, 50.0%) were for the 1999–2003 period while the least (*n* = 1714, 10.2%) was reported for the most recent five-year period (2014–2018).

**Table 1:** Papers that reported more than 100 deaths from poison

Year (mid study period)	location	Type of poisoning	Number of Deaths	Paper
2003	Bhopal	Aluminium phosphide	1455	[19]
2001	Rewa	Aluminium phosphide	112	[54]
2001	Rewa	Aluminium phosphide	122	[53]
2001	Chandigarh	Aluminium phosphide	359	[21]
1999	Chandigarh	Aluminium phosphide Insecticide	327 127	[22]
1999	Chandigarh	Aluminium phosphide	595	[24]
2000	Ujjain	Organophosphate	117	[27]
2000	West Bengal	Pesticide	148	[30]
2002	Karnataka	Insecticide	286	[34]
2002	Karnataka	Organophosphate	187	[35]
1999	Yavatmal	Pesticide Organochlorine	526 145	[40]
1999	Andra Pradesh	Pesticide	228	[47]
1998	Tamil Nadu	Pesticide	961	[44]
2001	Tamil Nadu	Pesticide	258	[45]
2007	Jhansi	Hair dye (paraphenylenediamine (PPD)	311	[71]
2005	Surat,	Pesticide	116	[74]
2004	Yavatmal	Insecticide	103	[78]
2008	Tamil Nadu	Pesticide	149	[81]
2008	Chandigarh	Aluminium phosphide	449	[91]
2010	Lucknow	Carbamate	110	[112]
2012	Himachal Pradesh	Pesticide	390	[123]
2013	Karnataka	Organophosphate	245	[137]
2012	Karnataka	Organophosphate	100	[140]
2012	Kerala	Organophosphate	219	[141]
2012	Maharashtra	Organophosphate	141	[157]
2018	Punjab	Aluminium phosphide	403	[169]
2016	Kanpur	Organophosphate Organochlorine	139 104	[177]
2015	Valsad	Organophosphate	106	[179]
2014	Tamil Nadu	Organophosphate	118	[185]

**Fig. 2:**
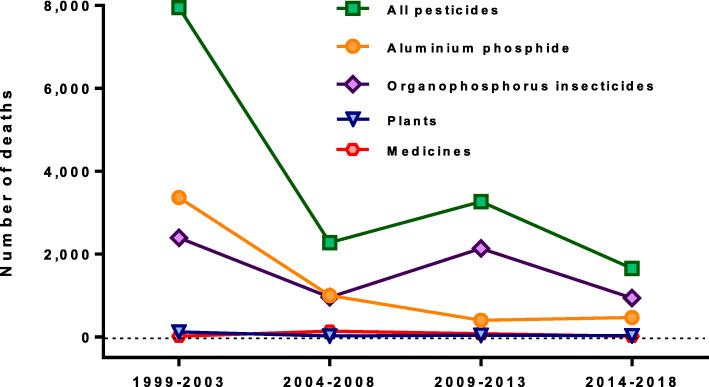
Reported deaths from poisoning by poison class in India by five-year period, 1999–2018

Most reported deaths (*n* = 15,748, 94.5%) were due to pesticides, of which OP insecticides (*n* = 6387, 40.5%) and aluminium phosphide (*n* = 5534, 35.1%) were identified as being responsible for 75%. Some papers classified deaths as only insecticides (*n* = 741) or pesticides (*n* = 1806), preventing more specific identification of the causative poison. Aluminium phosphide was the most important poison in the first five-year period (3363 deaths) but then rapidly fell in importance, being reported in just 461 deaths in the last period (Fig. 2). OP insecticides were important throughout the two decades, being responsible for 2541 deaths in the first 5 years and 940 in the last 5 years. Unfortunately, relatively few papers reported the individual OP insecticides involved but commonly reported compounds included monocrotophos [27, 73, 82, 111, 116] methyl parathion [38, 39, 81, 198], dimethoate [187], chlorpyrifos [187], quinalphos [141], dichlorvos [111], and phorate [158].

Organochlorine (*n* = 620), carbamate (*n* = 244) and pyrethroid (*n* = 25) insecticides, the herbicide paraquat (*n* = 305), rodenticides (*n* = 73), and fungicides (*n* = 14) were other pesticide classes that were identified as causing deaths. The most important organochlorine insecticide was endosulfan [56, 98] (after the ban of endrin in 1990 [203]). There were 233, 148, 101, and 138 deaths reported from organochlorines during the five periods, without any change in the last quarter after the nationwide ban of endosulfan in 2011. The paraquat deaths predominantly occurred during the 2009–2013 period (*n* = 167, 54.7% of total) with very few (*n* = 6, 1.9%) reported prior to 2009.

Hair dye poisoning was the cause of 439 deaths (2.6% of all reported deaths) during the middle decade, with 319 reported during 2004–2008 and 120 during 2009–2013. All were due to paraphenylenediamine (PPD) poisoning [77, 126–129, 166], either in the generic form of stone hair dye or the branded Vasmol 33. It is unclear whether cases continued after 2013, in the absence of reports; no regulatory activities were identified to explain a reduction.

Other chemicals, such as kerosene and household chemicals (eg. bleach), caused 62 deaths during the study period.

Deaths reported as being due to medicines or toxic plants were substantially less common than pesticide poisoning. There were 240 deaths due to medicines, accounting for 1.4% of deaths. The medicines noted were anti-tuberculosis drugs (*n* = 62), barbiturates (*n* = 14), dextropropoxyphene (*n* = 11), dapsone (*n* = 3), and alternative/complementary medicines (*n* = 3).

Toxic plants were responsible for 201 deaths, with the number markedly higher in the first 5-year period (1999–2003: 112; 2004–2008: 21; 2009–2013: 41; 2014–2018: 27). *Cleistanthus collinis* (oduvan, *n* = 131), *Cerbera odollam* (sea mango, *n* = 52), and *Cascabela thevetia* (yellow oleander, *n* = 16) were the common plant poisons recorded to have caused deaths.

Aluminium phosphide poisoning had a marked geographical distribution. Of the 38 papers reporting it as the primary cause of deaths, 35 (92.1%), reporting 4837 (99.4%) deaths, were from northern Indian states (Haryana, Punjab, Uttar Pradesh, Gujarat, Madhya Pradesh). Only 3 reports (with just 27 deaths) were from the south, all from Karnataka state. An additional seven papers reported aluminium phosphide as a secondary cause of death in the south (Karnataka, Maharashtra, Tamil Nadu) but again only 124 deaths were included. Overall, 97.2% (5079 deaths) of aluminium phosphide deaths occurred in the north compared to 2.8% (*n* = 146) in the south. This geographical distribution is due to this fumigant’s use for preservation of wheat grain which is predominantly grown in the north [204].

In contrast, the total number of deaths from poisons other than aluminium phosphide was significantly higher in southern states of Andhra Pradesh, Maharashtra, Telangana, Tamil Nadu, Karnataka, Kerala, and Orissa, than the rest of the country (*n* = 6747, 75.5%) vs (*n* = 2187, 24,5%) (Table 2).

**Table 2:** Number of deaths reported in publications from each State by five-year period, 1999–2018

State	1999–2003	2004–2008	2009–2013	2014–2018
Andhra Pradesh +Telangana	937	146	190	25
Capital Territory of Delhi	23	102	107	7
Gujarat	28	220	80	140
Haryana	1286	449	73	4
Karnataka	730	315	639	118
Kerala	46	57	239	9
Madhya Pradesh	1614	90	0	37
Maharashtra	1052	249	333	69
Other	56	15	525	64
Punjab	165	178	339	403
Tamil Nadu	1338	240	96	151
Uttar Pradesh	21	394	146	182
West Bengal	208	4	112	18

The distribution of plant poisoning was also geographically restricted with cases of *C collinis*, *C odollam*, and *C thevetia* poisoning all being reported from the south, especially Tamil Nadu and Kerala. Except for one large study reporting 311 deaths in Uttar Pradesh, all the studies reporting deaths from hair dye poisoning were from the southern states of Andhra Pradesh and Tamil Nadu.

## Discussion

Poisoning has been a major clinical and public health problem in LMIC, including India, for decades although the burden has often been ignored [7]. Despite the underreporting of poisoning deaths in India due to weaknesses in death certification, stigma associated with suicide [205], and lack of clinical and laboratory services to aid diagnosis, we found an alarming number of deaths in the Indian literature. The vast majority were due to intentional pesticide poisoning, indicating the importance of regulating highly hazardous pesticides (HHPs) to prevent deaths as recommended by the WHO and FAO [206–209]. Continued global regulation of pesticides to remove HHPs from poor smallholder farms in LMICs, which are totally unable to use them safely, will rapidly reduce pesticide and total suicide rates [210, 211].

PIC data is useful for understanding the epidemiology of cases presenting to hospital, but cannot be used to assess the burden of poisoning deaths in India. PICs do not routinely collect data on the outcome of patients after a phone consultation. A prospective PIC call study in Sri Lanka showed that mortality was < 10% among cases reported to PIC [212]. Many of the patients discussed with PIC were not critically ill, with advice frequently being sought for mildly poisoned patients [213]. PICs do not get called about all poisoning patients and frequent inquiries about one particular poison does not mean that it’s a common cause of poisoning death in the community. The Sri Lankan national PIC receives 315 calls/year on average while poisoning hospital admissions exceed 80,000 – a 250-fold greater number of cases [212]. In Switzerland, only 11.4% of poisoned patients were referred to the PIC [213]. PICs are usually utilised for identification or management of unfamiliar poisonings and, even for these unusual presentations, information via telephone may not be a clinician’s first choice. Doctors may be reluctant to seek assistance from non-clinicians in the PIC and prefer the internet over the telephone in obtaining such poisons information [214]. The availability of an online database (TOXBASE) in the United Kingdom has caused a dramatic decline in the telephone enquires [215]. The utilisation of web based resources for such poison related information will affect the representativeness of PIC data [216].

This Indian literature review provides some interesting information. Aluminium phosphide self-poisoning was first reported in north Indian states around Delhi in the 1980s [217–219] before becoming a major practically untreatable clinical problem with no antidote [18, 220]. Remarkably, a change in the formulation of the fumigant in 2001, removing the 3 g 56% aluminium phosphide tablets from open sale [203], has resulted in a major reduction in deaths reported in the literature. This is supported by the single hospital study performed at Nehru Hospital, Chandigarh, in which the case fatality fell from 59% to 22% [17]. This appears to have been a great public health and pesticide regulatory success that could be replicated for OP insecticides. However, the situation seems to be different in Iran [221]. Despite a ban of aluminium phosphide tablets in 2007, fatal cases continued to be common in Iran. The incidence of fatal aluminium phosphide cases referred for phosphine analysis in Tehran increased from 5.2 per million population in 2006 to 37.0 in 2013. Aluminium phosphide was reported as being responsible for 2007 deaths during these 8 years, 85% from self-poisoning [221].

Deaths from OP insecticide poisoning were common throughout the study period, being only modestly less important than aluminium phosphide at the beginning and far more important at the end. The experience of Sri Lanka [222–224] and Bangladesh [225] has shown that bans of highly hazardous OP insecticides result in a rapid fall in pesticide suicides, without apparent effect on agricultural productivity [211, 225–227]. The Indian government banned the key OP insecticide parathion in 1974 after recognising its important role in fatal poisoning [203, 228]. Monocrotophos was banned from vegetable production in 2005; however, this restriction is not widely enforced and it remains commonly used in small scale agriculture [229, 230] and therefore self-harm.

The Indian government banned 18 pesticides in 2018 and 2020 [231], including several highly hazardous OP insecticides used for self-harm (fenthion, methyl parathion, dichlorvos, phorate, phosphamidon, and triazophos). These regulations have the potential to markedly reduce deaths from poisoning across India; its effects on suicide, occupational poisoning and agricultural output needs to be carefully monitored. The Government has recently proposed banning another 27 pesticides, including monocrotophos and dimethoate which are acutely toxic pesticides when used for self-poisoning.

The organochlorine insecticide endrin was responsible for many deaths in the 1960s–80s until being banned in 1990 [203]. Endosulfan was thereafter a major problem until it too was banned in Kerala in 2005 and across India in 2011; the effect of this ban is unclear from these data since the number of cases reported during the last 10 years remained quite steady. Illegal importation of some endosulfan, despite the ban, might be partially responsible [232]. Organochlorine insecticide deaths are still in reported in some states of India (Rajasthan, Andhra Pradesh, Karnataka, Tamil Nadu) [233]. The effects of the endosulfan ban, driven by the Indian Supreme Court [234], should be carefully studied, although initial analyses using national police data suggest that it may have been associated with a national fall in pesticide suicides [203].

We found relatively few deaths from herbicides, including paraquat; they were responsible for just 1.8% of all reported pesticide deaths. This runs counter to claims that herbicides have been commonly taken in suicide attempts in India [100]. However, there was an increasing trend in paraquat poisoning in India. A hospital based study performed during 1999–2006 reported five patients (three deaths) in Punjab [28], while a second from Tamil Nadu reported ten patients with 100% mortality [235]. A recent newspaper report states that paraquat poisoning is a problem in Orissa with more than 100 deaths reported during 2018 and 2019 [236]. If paraquat becomes widely used for agriculture and self-harm in India, there is the risk of a massive increase in deaths as occurred in China before its ban in 2016.

Fatal plant poisoning with *C collinus*, *C odallam*, and *C thevetia* were relatively uncommon. The number was greatest in the first 5 years of study, before falling steadily by 75%. Most cases were suicides and occurred in rural regions of south Indian states. It is possible that medical therapy has improved with experience.

Medicine poisoning deaths are not uncommon in high income countries, especially where medical opioid addiction is widespread [237]. An increase in the number of cases of medicine poisoning has been reported from urban Sri Lanka [238] but we found few deaths in this study, likely due to the relatively low case fatality of medicine poisoning [7]. The incidence of medical opioid addiction currently seems low in India [239].

Dextropropoxyphene has long been used as an analgesic, alone or in combination with paracetamol (acetaminophen). However, many European countries including England and Scotland banned its use during 2005–2009 due to its importance for suicide [240]. Based on these findings, the central government of India suspended its manufacture, sale and distribution in 2013 [240]. The deaths from dextropropoxyphene reported in this study occurred in 2011–2012 [113], before its ban.

### Limitations

The use of the published literature from multiple hospitals over 20 years to get a better understanding of the epidemiology of lethal poisoning in India has clear limitations. Secular variation in publishing practices and interest in poisoning possible are two of them. The best approach would be to have a network of sentinel hospital sites across India collecting prospective data and reporting their experience with both hospital cases and poisoning deaths that occur in their communities, before hospital admission, as recommended by the WHO [241]. This would allow for systematic collection of data that should allow interpretation of changes over time, especially in response to regulatory changes. Unfortunately, there is no network of such sentinel sites in India, although a few hospitals have reported their experience over the medium term (for example the Nehru Hospital, Chandigarh).

A further key limitation is that our review is based on papers in English and available online, which means we will have missed studies in other Indian language and papers that were not available online. A number of studies reporting results in other Indian languages did have abstracts in English, but this lacked the detailed information required for the review. However, the large number of studies found (186 papers, 16,659 deaths) suggests that these additional papers are unlikely to have changed its key conclusions.

All the studies included here were either from a healthcare facility or a forensic autopsy study. Therefore, patients who die before reaching hospitals may not be fully represented in these studies (although forensic case series usually include deaths occurring pre-hospital). The importance of under-reporting of suicides is clearly shown by verbal autopsy studies [242] which show markedly higher rates of suicide than reported in government statistics. It is therefore possible that a fast-acting poison, which kills people before they get to hospital, may be under-represented in this analysis. Many studies were of hospitalised patients in a few selected institutes; the vast majority of hospitals seeing such patients do not publish their experience, suggesting some bias. Many poisoning patients leave hospital against medical advice preventing any follow up, resulting in underreporting of some deaths. Extrapolating police records on poisoning autopsies and poisoning death data from forensic studies would be useful to gain a fuller picture of poisoning deaths in India.

Since we wished to get a broad-brush picture of lethal poisoning, we did not include every lethal case in the papers identified but only the two most common causes. This means that relatively uncommon poisons such as plants and medicines will be under-represented in the total picture. However, in very few papers did these poisons make up more than 5% of the lethal cases and so including all the deaths in all 186 papers would unlikely have changed the conclusions.

For the above reasons, in particular the lack of data on prevalence or incidence, as well as population and region, the data did not allow a State- or period-based calculation of the incidence of lethal poisoning or for a sex- and age-based analysis. The number of cases reported in the literature is likely to be a small sub-sample of all deaths occurring. Indeed, Patel and colleagues estimated that poisoning accounts for about 70,000 deaths every year in India [5] compared to the 833 deaths/year reported in this systematic review.

## Conclusion

In this study of the literature, we found that pesticide poisoning was a major cause of death in India throughout the study period, although aluminium phosphide appears to have become much less important after government regulation. Similar government regulation is underway for OP insecticides, which could have a major effect on the number of poisoning deaths over the next few years. Deaths from plant and medicine poisoning remain uncommon.

## Data Availability

All data are available online as given in the reference list.

## Abbreviations

WHO: World Health Organisation
LMIC: Lower- and Middle-Income Countries
HHP: Highly Hazardous Pesticides
OP: Organophosphorus
PIC: Poison Information Centre
FAO: Food and Agriculture Organisation
